# Exogenous GM-CSF therapy for autoimmune pulmonary alveolar proteinosis: a systematic literature review

**DOI:** 10.3389/fmed.2025.1552566

**Published:** 2025-05-08

**Authors:** Wushu Chen, Xin Feng, Lun-kai Yao, Xingpei Li, Zhen-ming Yang, Xiu-yu Qin, Yu Li, Ye Qiu

**Affiliations:** ^1^Gastroenterology and Respiratory Internal Medicine Department, Guangxi Medical University Cancer Hospital, Nanning, Guangxi, China; ^2^State Key Laboratory of Respiratory Disease, National Clinical Research Center for Respiratory Disease, Guangzhou Institute of Respiratory Health, The First Affiliated Hospital of Guangzhou, The First Affiliated Hospital of Guangzhou Medical University, Guangzhou, China; ^3^Medical Oncology of Respiratory Medicine, Guangxi Medical University Cancer Hospital, Nanning, Guangxi, China; ^4^Department of Respiratory and Critical Medicine, Yiyang Central Hospital, Yiyang, Hunan, China

**Keywords:** GM-CSF, aPAP, inhalation, subcutaneous injection, treatment dosage and duration

## Abstract

**Background:**

Granulocyte-macrophage colony-stimulating factor (GM-CSF) therapy is an important treatment for autoimmune pulmonary alveolar proteinosis (aPAP). Exogenous GM-CSF treatment can be administered either through subcutaneous injection or nebulized inhalation. However, data on the effectiveness and safety of these two approaches are lacking.

**Method:**

We conducted a systematic literature review of different methods, including subcutaneous injection and nebulized inhalation of GM-CSF, for the treatment of aPAP patients. Patients were divided into a subcutaneous injection group (SIG) and a nebulized inhalation group (NIG) according to the route of administration. Treatment efficacy and safety, including adverse events, were statistically assessed. We analyzed different GM-CSF treatment cycles with different time intervals. The analyses were performed using chi-square tests, unpaired *t*-tests, and Kruskal–Wallis *H*-tests.

**Results:**

A total of 304 aPAP patients were treated with GM-CSF, including 66 (21.7%) in the SIG and 238 (78.3%) in the NIG. In total, we identified 220 (72.37%) patients whose treatment was effective and 84 (27.63%) patients whose treatment was ineffective. Efficacy was achieved in 54.55% (36/66) of the SIG patients and 77.31% (184/238) of the NIG patients (*P* < 0.001). More metrics were changed than in the NIG than SIG, suggesting the superior effectiveness of nebulized inhalation. The nebulized inhalation of GM-CSF was more effective (*P* < 0.001) and caused fewer adverse events than its subcutaneous injection. A significant difference in the NIG was noted across treatment durations, with an efficacy rate of 88% for those treated for over 24 weeks, compared with 48% in the SIG (*P* < 0.001). Among the NIG patients, the optimal efficacy was found to be at a dosage of 300–400 μg/d, with diminishing efficacy at higher doses (*P* < 0.036).

**Conclusion:**

Nebulized inhalation is a more effective and safer route of GM-CSF administration than subcutaneous injection is, with a potential optimal dosage of 300–400 μg/day, and the duration of GM-CSF treatment via nebulized inhalation with the greatest efficacy is >24 weeks.

## Introduction

Pulmonary alveolar proteinosis (PAP) is a rare disorder characterized by surfactant accumulation in the alveoli, leading to impaired gas exchange, progressive dyspnoea, and respiratory failure (1). The estimated PAP incidence is 6.87 ± 0.33 per million individuals, which is likely underestimated due to diagnostic challenges (2). Primary PAP, the most common form, includes autoimmune PAP (aPAP), which is defined by circulating anti-granulocyte-macrophage colony-stimulating factor (GM-CSF) antibodies and constitutes more than 90% of cases (1, 3).

No drugs are approved as therapy for aPAP in any country, and whole-lung lavage (WLL) is a key treatment for PAP, effectively improving oxygenation and reducing symptoms by clearing surfactant accumulation from the alveoli (4). It offers durable symptom relief as a form of palliative care, but its invasive nature, requiring general anesthesia and mechanical ventilation, poses risks such as infection and pneumothorax. Additionally, WLL is only available in specialized centers and may need to be repeated over time, as it does not address the underlying cause of PAP (5). Therefore, we need to find an appropriate medication regimen to address this issue.

GM-CSF is a promising treatment for aPAP, as it restores alveolar macrophage function, improves surfactant clearance and gas exchange, and reduces the need for WLL (4, 6, 7). GM-CSF is a glycoprotein that stimulates the production and function of white blood cells, including granulocytes and macrophages (8). In the context of aPAP, GM-CSF increases the activity of alveolar macrophages, which are responsible for clearing excess surfactant from the alveoli. In aPAP, antibodies inhibit the effects of GM-CSF, leading to impaired macrophage function and surfactant accumulation. Administering GM-CSF can help restore macrophage function (9).

Currently, GM-CSF treatments can be administered either through subcutaneous injection or nebulized inhalation (10, 11). However, there is a lack of comparative studies between these two approaches, and the optimal dose and treatment duration remain undefined.

Therefore, we designed this study to investigate the impact of subcutaneous injection vs. nebulized inhalation on treatment outcomes and to identify an appropriate dose range.

## Materials and methods

### Systematic literature review and search strategy

We conducted a systematic literature review of the efficacy of different methods of GM-CSF infusion in patients with aPAP. We searched for articles on aPAP published in English from January 1, 1985, to May 31, 2024, through databases such as PubMed, Web of Science, Embase, the BIOSIS Library, and the CNKI and WANFANG databases.

### Data filtering and extraction

Our selection requirements for the retrieved articles included the following: (1) the selected articles were limited to publications published in English; (2) the patients in the selected articles were patients with PAP and were treated with GM-CSF therapy; and (3) articles on diagnostics, immunologic and experimental studies, Additionally, duplicate cases were excluded, and general reviews were filtered. We extracted the following information from the patients in the selected articles: sex, age, geographic region, smoking history, PAP typing, GM-CSF treatment, treatment period, treatment dose, efficacy, toxicity, and pre- and post-treatment baseline, including forced expiratory volume in 1 s (FEV1), forced expiratory volume in 1 s/forced vital capacity (FEV1/FVC), and carbon monoxide diffusing capacity (DLCO) baseline.

### Grouping

We divided the patients into a subcutaneous injection group (SIG) and nebulized inhalation group (NIG) according to the route of administration.

### Efficacy

The effect of GM-CSF treatment on the collected patients was statistically analyzed. Markers for effective aPAP treatment include significant increases in PaO_2_ and SaO_2_ values and a reduced (A-a) O_2_ gradient, indicating enhanced oxygenation and gas exchange. An increase in DLCO suggests better diffusing capacity, whereas improvements in FEV1, FVC, total lung capacity (TLC), and vital capacity (VC) reflect improved lung function and increased lung volume. Radiological findings, such as reduced ground-glass opacities on high-resolution computed tomography (HRCT), further confirm treatment efficacy. Additionally, a decrease in the total cell count in bronchoalveolar lavage (BAL) fluid indicates a reduction in alveolar protein accumulation. Together, these markers are indicative of a positive response to treatment. Treatment was considered effective if these markers were observed in the patients. Treatment was considered ineffective if the disease symptoms were not alleviated or if the patient subsequently relapsed or stopped treatment for any reason.

### Safety

The safety of the included patients was analyzed. Safety included the occurrence of treatment-related adverse events and some sudden physical events. These events included skin symptoms, soreness, night sweats, exhaustion symptoms, digestive symptoms, eye symptoms, muscle and bone symptoms, respiratory symptoms, thermoregulatory symptoms, chest symptoms, psychological symptoms, hematological symptoms, urinary symptoms, pregnancies, weight increase, progression of aPAP, and swelling.

### GM-CSF treatment cycle and dose

We collected information on the number of GM-CSF treatment cycles and categorized the patients on the basis of this information. We also collected information on the dose of GM-CSF administered to each patient, and for the convenience of statistical analysis, we standardized the unit to kg administered per day by setting the patient weight to 60 kg and converting the dose administered per day on the basis of this weight.

### Statistical analysis

The data were analyzed via Statistical Products and Services Solutions (SPSS, version 25), and *P-*values were calculated via the chi-square test, unpaired *t*-test and Kruskal–Wallis *H*-test. *P-*values < 0.05 were considered significant.

## Results

**Figure 1:**
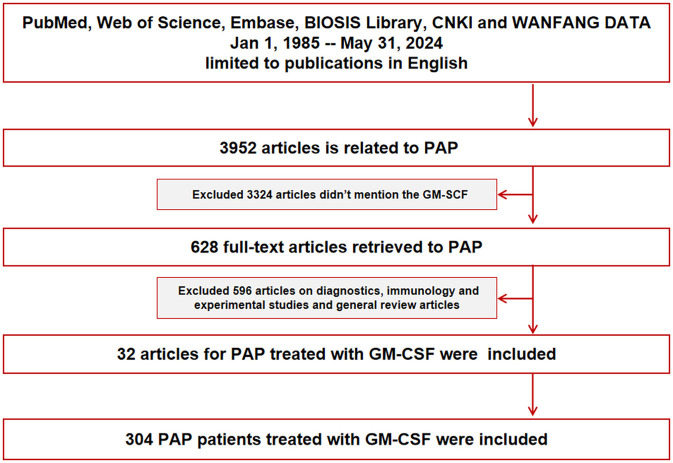
Flowchart of the collection process.

We searched 3,952 articles related to PAP on PubMed, Web of Science, Embase, the BIOSIS Library, and the CNKI and WANFANG databases and ultimately selected 32 English publications from Jan 1, 1985–May 31, 2024, on GM-CSF-treated patients with aPAP. A total of 304 aPAP patients treated with GM-CSF were examined in this study, including 66 (21.71%) patients in the SIG and 238 (78.29%) patients in the NIG (Figure 1).

### Patient characteristics

The analysis revealed a mean age of 39.92 years in the SIG and 50.72 years in the NIG (*P* < 0.001). Compared with the NIG, the SIG had more males (75.80%) (54.60% males, 45.40% females), with a significant sex difference (*P* = 0.002). The percentages of patients from different regions were significantly different between groups: patients from Asia (7.58% vs. 84.45%, *P* < 0.001), patients from North America (68.18% vs. 7.56%, *P* < 0.001), and patients from Australia/Europe (24.24% vs. 7.99%, *P* < 0.001). The percentage of patients that had aPAP for 2–3 years (74.24% vs. 6.72%, *P* < 0.001) and >3 years (10.61% vs. 2.10%, *P* = 0.005) were different between groups. The percentages of nonsmokers (12.12% vs. 24.79%, *P* = 0.028) and ex-smokers (36.36% vs. 15.97%, *P* = 0.036) were significantly different between groups. The percentages of patients with previous lavage counts of 1–5 (60.61% vs. 31.93%, *P* < 0.001) and 5–10 (13.64% vs. 2.94%, *P* = 0.02) were significantly different between groups. Significant differences in the percentages of patients with high bronchoalveolar lavage fluid (BALF) protein levels (3.03% vs. 62.61%, *P* < 0.001), of patients who underwent open lung biopsy (OLB) (28.79% vs. 53.36%, *P* = 0.001), of patients who underwent transbronchial biopsy (TBB) (66.67% vs. 6.72%, *P* < 0.001), and who had high levels of anti-GM-CSF antibodies (1.52% vs. 83.19%, *P* < 0.001) were present between the two groups (Table 1).

**Table 1:** Baseline data.

**Variable**	**Subcutaneous injection *n* (%)**	**Nebulized inhalation *n* (%)**	***P-*value**
Number	66	238	
**Gender**			0.002
Male	50 (75.80%)	130 (54.60%)	
Female	16 (24.20%)	108 (45.40%)	
Age	39.92 ± 8.95	50.72 ± 8.13	<0.001
**Region**			
Asian	5 (7.58%)	201 (84.45%)	<0.001
North America	45 (68.18%)	18 (7.56%)	<0.001
Australia and Europe	16 (24.24%)	19 (7.99%)	<0.001
**PAP course**			
< 2 years	10 (15.15%)	41 (17.23%)	0.690
2–3 years	49 (74.24%)	16 (6.72%)	<0.001
>3 years	7 (10.61%)	5 (2.10%)	0.005
**Smoking history**			
Smokers	28 (42.42%)	72 (23.95%)	0.063
Non-smokers	8 (12.12%)	59 (24.79%)	0.028
Ex-smokers	24 (36.36%)	56 (15.97%)	0.036
**Diagnosis**			
BALF	2 (3.03%)	149 (62.61%)	<0.001
OLB	19 (28.79%)	127 (53.36%)	0.001
Transbronchial lung biopsy (TBLB)	0	11 (4.62%)	0.160
TBB	44 (66.67%)	16 (6.72%)	<0.001
Thoracoscopic lung biopsy	2 (3.03%)	1 (0.42%)	0.120
Anti-GM-CSF antibodies	1 (1.52%)	198 (83.19%)	<0.001
**GM-CSF pretreatment activity tolerance**			
<50 m	2 (3.03%)	19 (7.98%)	0.116
300–400 m	2 (3.03%)	41 (17.23%)	0.003
>400 m	2 (3.03%)	101 (42.44%)	<0.001
**Number of previous lavages**			
1–5 times	40 (60.61%)	76 (31.93%)	<0.001
5–10 times	9 (13.64%)	7 (2.94%)	0.02
>10 times	7 (10.61%)	18 (7.56%)	0.426

### Pre- and post-treatment comparisons

Pre- and post-treatment comparisons were made between the SIG and NIG. More metrics were altered after nebulized inhalation of GM-CSF than subcutaneous injection.

In the SIG, significant increases in PaO_2_ values (from 58.61 ± 8.93 to 79.36 ± 12.11 mmHg, *P* < 0.001) and SaO_2_ values (from 77.75 ± 11.32% to 93.00 ± 3.21%, *P* = 0.034) and significant decreases in (A-a) O_2_ values (from 40.93 ± 5.05 to 15.70 ± 4.13 mmHg, *P* < 0.001) were observed (Table 2).

**Table 2:** Pre- and post-treatment baseline data for different groups.

**Metrics**	**Pre-treatment *n* (%)**	**Post-treatment *n* (%)**	***P-*value**
**Subcutaneous injection group**			
PaO_2_ (mmHg)	58.61 ± 8.93	79.36 ± 12.11	<0.001
PaCO_2_ (mmHg)	59.59 ± 7.97	42 (*n =* 1)	0.066
SaO_2_	77.75 ± 11.32%	93.00 ± 3.21%	0.034
FVC baseline	78.09 ± 7.60%	85.26 ± 8.85	0.165
FEV1	69.66 ± 23.69%	91% (*n =* 1)	0.655
TLC	64.00 ± 18.38%	81% (*n =* 1)	0.211
VC	55.00% (*n =* 1)	99.00% (*n =* 1)	0.317
VO_2max_ maximal oxygen uptake (ml/kg/min)	16 (*n =* 1)	28 (*n =* 1)	-
DLCO	38.00 ± 9.90%	66 ± 2.83%	0.121
(Aa) O_2_ baseline (mmHg)	40.93 ± 5.05	15.70 ± 4.13	<0.001
BAL Protein Levels (g/L)	2 (*n =* 1)	0.2 (*n =* 1)	-
Total number of BAL cells (cell/L)	600 (*n =* 1)	4^*^10^4^ (*n =* 1)	-
**Inhalation group**			
PaO_2_ (mmHg)	63.93 ± 5.85	77.43 ± 5.56	<0.001
PaCO_2_ (mmHg)	37.74 ± 1.80	30.3 ± 2.54	0.013
SaO_2_	84.95 ± 2.98%	89% (*n =* 1)	0.008
FVC baseline	79.97 ± 7.27%	84.98 ± 4.20	<0.001
FEV1	83.43 ± 9.24%	86.83 ± 5.33%	0.002
FEV1/FVC	86.06 ± 2.82%	85.11 ± 1.15%	0.045
TLC	61.72 ± 11.62%	85.83 ± 7.85%	<0.001
VC	77.36 ± 4.41%	82.51 ± 3.47%	<0.001
VO_2max_ maximal oxygen uptake (ml/kg/min)	19.9 (*n =* 4)	26.3 (*n =* 4)	-
DLCO	53.54 ± 8.52%	61.92 ± 8.66%	<0.001
(Aa) O_2_ baseline (mmHg)	41.26 ± 6.02	27.79 ± 5.10	<0.001
Total number of BAL cells (cell/L)	19.1 ± 3.2^*^10^(n = 19)^	29.0 ± 4.8^*^10 (*n =* 19)	<0.001

In the NIG, the PaO_2_ value increased from 63.93 ± 5.85 to 77.43 ± 5.56 mmHg (*P* < 0.001), the PaCO_2_ value decreased from 37.74 ± 1.80 to 30.3 ± 2.54 mmHg (*P* = 0.013), and the FEV1 value increased from 83.43 ± 9.24% to 86.83 ± 5.33% (*P* = 0.002). Significant changes were also observed in TLC (from 61.72 ± 11.62% to 85.83 ± 7.85%, *P* < 0.001), VC (from 77.36 ± 4.41% to 82.51 ± 3.47%, *P* < 0.001), DLCO (from 53.54 ± 8.52% to 61.92 ± 8.66%, *P* < 0.001), and (A-a) O_2_ values (from 41.26 ± 6.02 to 27.79 ± 5.10 mmHg, *P* < 0.001) (Table 2).

We also compared the differences in metrics before and after treatment and detected differences in the metrics PaO_2_ (*P* < 0.001), VC (*P* = 0.015), DLCO (*P* < 0.001), and (A-a) O_2_ baseline (mmHg) (*P* < 0.001) (Supplementary Table 1).

### Efficacy

In total, we identified 220 (72.37%) patients whose treatment was effective and 84 (27.63%) patients whose treatment was ineffective. Our analysis revealed that 36 (54.55%) patients in the SIG were effectively treated, whereas 184 (77.31%) patients in the NIG were effectively treated (*P* < 0.001) (Table 3).

**Table 3:** Efficacy.

**Treatment method**	**Effective**	**Ineffective**	***P-*value**
Subcutaneous injection	36 (54.55%)	30 (44.45%)	<0.001
Nebulized inhalation	184 (77.31%)	54 (22.69%)	

### Safety

**Figure 2:**
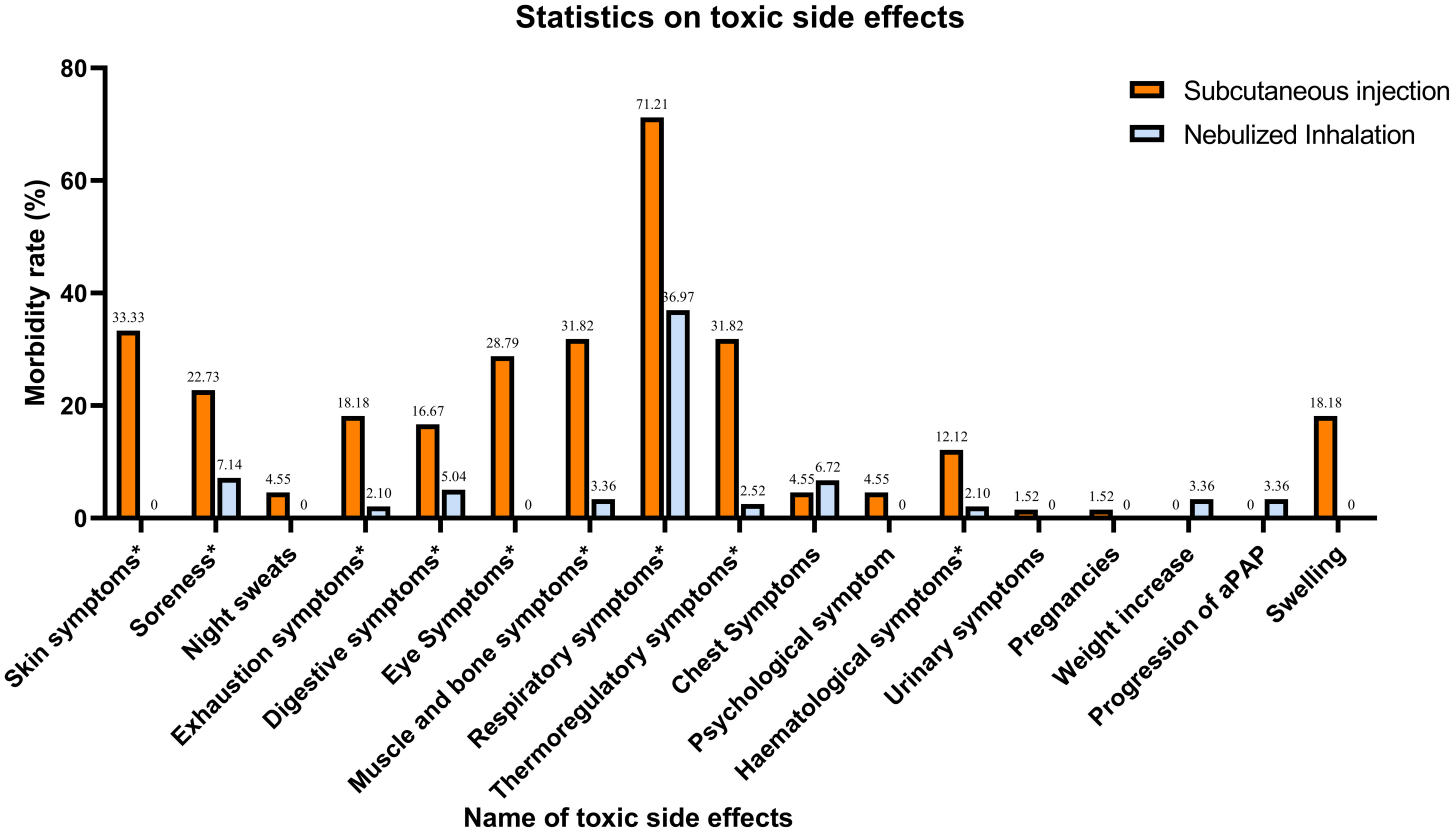
Statistics on the concomitant toxic effects of treatment. Symptoms with * are those where there is a significant difference in toxicity between the two treatment methods (*P* < 0.005).

In terms of safety, different aspects of toxicity were observed in both the SIG and NIG (Table 4, Figure 2). There were significant differences between the SIG and NIG for various symptoms. Skin symptoms occurred in 33.33% of the SIG patients but not in the NIG patients (*P* < 0.001). Soreness was observed in 22.73% of the SIG vs. 7.14% of the NIG patients (*P* = 0.001). Exhaustion symptoms were reported by 18.18% of the SIG patients and 2.10% of the NIG patients (*P* < 0.001). Digestive symptoms were observed in 16.67% of the SIG patients and 5.04% of the NIG patients (*P* = 0.003). Eye symptoms were present in 28.79% of the SIG patients but not in the NIG patients (*P* < 0.001). Swelling occurred in 18.18% of the SIG patients (*P* < 0.001), whereas muscle and bone symptoms were more common in the SIG patients (31.82% vs. 3.36%, *P* < 0.001). Respiratory symptoms were more common in the SIG (71.21%) patients than in the NIG patients (36.97%, *P* < 0.001). Thermoregulatory symptoms were observed in 31.82% of the patients in the SIG and 2.52% of those in the NIG (*P* < 0.001). Hematological symptoms were more common in the SIG patients (12.12%) than in the NIG patients (2.10%, *P* = 0.002).

**Table 4:** Safety.

**Name of toxic side effect**	**Subcutaneous injection**	**Nebulized inhalation**	***P-*value**
Skin symptoms	22 (33.33%)	0 (0%)	<0.001
Soreness	15 (22.73%)	17 (7.14%)	0.001
Night sweats	3 (4.55%)	0 (0%)	0.11
Exhaustion symptoms	12 (18.18%)	5 (2.10%)	<0.001
Digestive symptoms	11 (16.67%)	12 (5.04%)	0.003
Eye symptoms	19 (28.79%)	0 (0%)	<0.001
Muscle and bone symptoms	21 (31.82%)	8 (3.36%)	<0.001
Respiratory symptoms	47 (71.21%)	88 (36.97%)	<0.001
Thermoregulatory symptoms	21 (31.82%)	6 (2.52%)	<0.001
Chest symptoms	3 (4.55%)	16 (6.72%)	0.353
Psychological symptom	3 (4.55%)	0 (0%)	0.11
Hematological symptoms	8 (12.12%)	5 (2.10%)	0.002
Urinary symptoms	1 (1.52%)	0 (0%)	0.223
Pregnancies	1 (1.52%)	0 (0%)	0.223
Weight increase	0 (0%)	8 (3.36%)	0.129
Progression of aPAP	0 (0%)	8 (3.36%)	0.129
Swelling	12 (18.18%)	0 (0%)	<0.001

### GM-CSF treatment cycle

**Figure 3:**
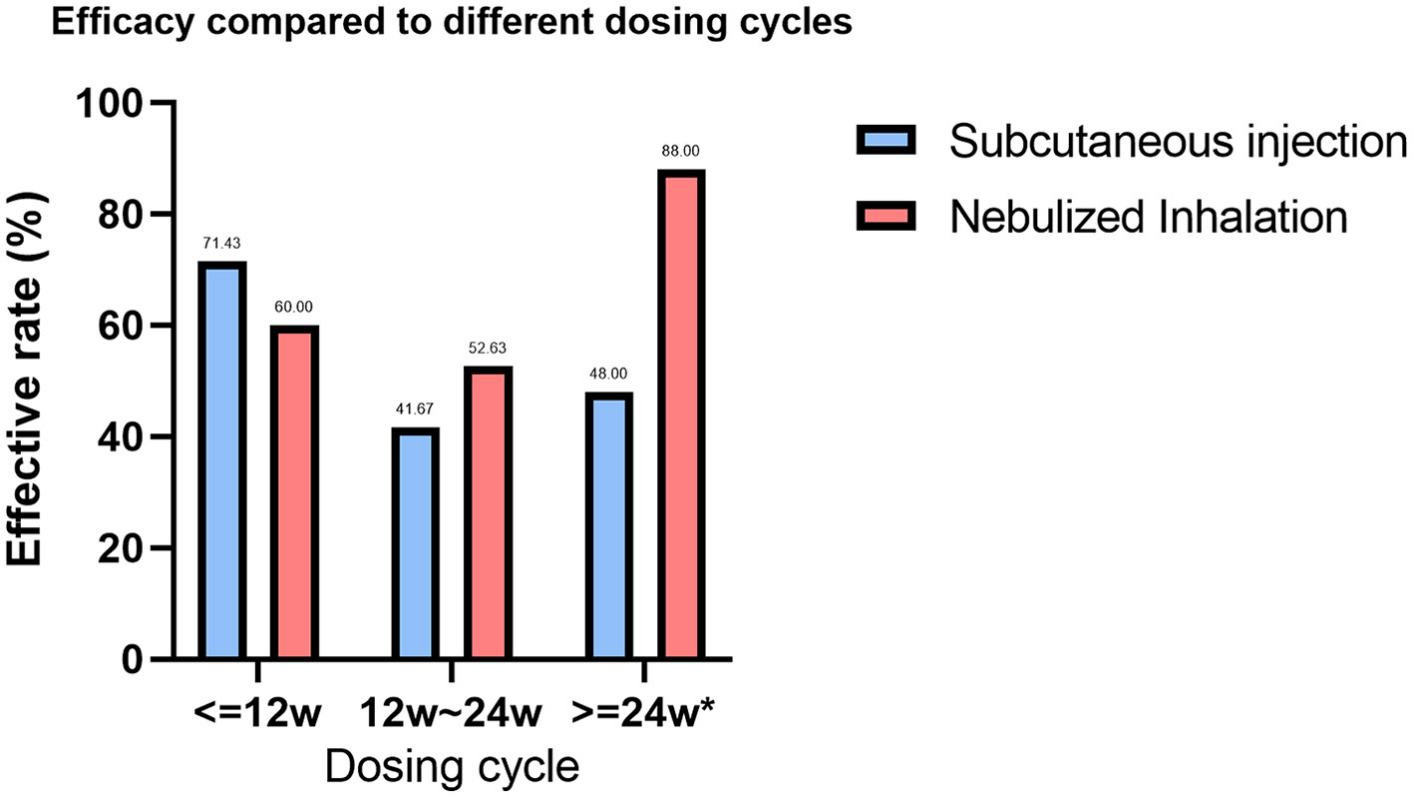
Comparison of the efficacy rates across treatment cycles for different GM-CSF delivery methods. Treatment cycles with * are defined as significantly different between the efficacy rates of the two GM-CSF treatments (*P* < 0.005).

In the statistical analysis of treatment cycles, 44 patients in the SIG had documented treatment cycles, with 22 (50.00%) having an effective treatment cycle and 22 (50.00%) having an ineffective treatment cycle. In the NIG, 89 patients had documented treatment cycles, of which 54 (60.67%) were effective and 35 (39.33%) were ineffective (*P* < 0.001). We analyzed the SIG and NIG treatment cycles separately, dividing treatment durations into ≤ 12 weeks, 12–24 weeks, and >24 weeks (Supplementary Table 2). A statistically significant difference was found between different treatment durations in the NIG (*P* = 0.031), where 25 patients had >24 weeks of treatment, with an effectiveness rate of 88%. Additionally, we compared the efficacy of different treatment cycles between the SIG and NIG (Supplementary Table 3, Figure 3). Among the patients whose treatment cycle was >24 weeks, 48% of SIG patients were effectively treated, whereas 88% in the NIG were effectively treated, indicating a significant difference (*P* < 0.001).

### GM-CSF dosage

In the subgroup analyses for the SIG, dosages such as <=400 mg/day and >400 mg/day (*P* = 0.795) did not significantly differ in terms of efficacy. However, inhalation dosages of <=400 mg/d were significantly effective (*P* = 0.036), whereas those of <=300 μg/d were significantly more effective (*P* = 0.076). Overall, nebulized inhalation appears to be a more effective administration route for GM-CSF than subcutaneous injection is (Supplementary Table 4).

## Discussion

Although WLL is currently the main treatment for PAP, it is important to keep in mind that, as suggested by Campo et al. (12), procedural differences between centers should be considered as they may have an impact on the treatment's safety and effectiveness. Variations in lavage volume, frequency, etc. are a few examples. Additionally, this serves as a reminder of how crucial protocol standardization is to maximizing WLL's contribution to PAP.

In our study, one of the largest studies to date on aPAP treatment with GM-CSF, 304 patients were analyzed and the efficacy and safety of GM-CSF subcutaneous injection vs. nebulized inhalation were compared. The findings revealed that inhalation is more effective and has a better safety profile, with fewer side effects and a stronger dose–response relationship, particularly at 300–400 μg/d. These insights are crucial for optimizing aPAP treatment strategies, emphasizing the superiority of inhalation therapy in achieving better patient outcomes. In addition, it is noteworthy that the results we found appear to be different from the findings of the previous PAGE trial (10) and the IMPALA trial (13) (250 μg/day in the former; 300 μg/day in the latter). This discrepancy could result from variations in pharmacokinetic variables, patient compliance, nebulizer efficiency, etc. For instance, the PAGE trial employed a jet nebulizer, which has a reduced deposition efficiency, but the MPALA trial used a vibrating mesh nebulizer, which improves medication delivery to the alveoli. This tells us that device-specific adjustments are also an important aspect of optimizing the therapeutic effect. And this echoes the findings of Luisetti et al. (14) that when using the AKITA^2^ APIXNEB^®^ Nebulizing System, ~49.9% of the administered dose was targeted for deposition in the alveolar region (total lung deposition rate of 80.4%, with a nominal dose of 250 μg corresponding to an alveolar dose of 96 μg). This finding highlights the central impact of delivery technology on efficacy. In addition, the investigators emphasized that the abnormal accumulation of surface-active substances in the alveoli of patients with PAP may significantly reduce the actual deposition efficiency. Therefore, higher nominal doses are required in the clinic to compensate for the pharmacokinetic losses in such pathological settings, while the risk of systemic exposure due to dose overruns needs to be guarded against. Further optimization of therapeutic regimens can be achieved in the future through accurate metrological modeling, the use of *in vivo* validation techniques, and the improvement of device capabilities and quality. In this research, we found that nebulized inhalation was more effective than subcutaneous injection was, which is consistent with findings from previous meta-analyses (15). We observed that a dosage of ≤ 400 μg/day showed better efficacy in the NIG, whereas no clear threshold was identified at ~300 μg/day in the SIG. This may be because elevated anti-GM-CSF antibody levels induce a suppressive state, whereas excessively high GM-CSF levels lead to hyperactivation, disrupting immune homeostasis. An optimal dose increases immune cell numbers, but excessive dosing can reduce cell counts (16, 17). Nebulized inhalation of GM-CSF is a promising and safe pharmacological strategy that restores alveolar macrophage function, promotes surfactant clearance, improves lung function in aPAP patients (6). Inhalation of GM-CSF is less invasive than subcutaneous injection, offering greater convenience and reduced discomfort for patients. This method ensures targeted delivery to the lungs, minimizing systemic side effects while increasing local efficacy (10, 13). Its prolonged presence in the lungs offers sustained stimulation of alveolar macrophages, potentially leading to more effective and enduring therapeutic outcomes (18, 19). Compared with subcutaneous injections, inhaled GM-CSF is less likely to cause systemic adverse effects, with a focus on its impact on the respiratory system (20, 21).

**Figure 4:**
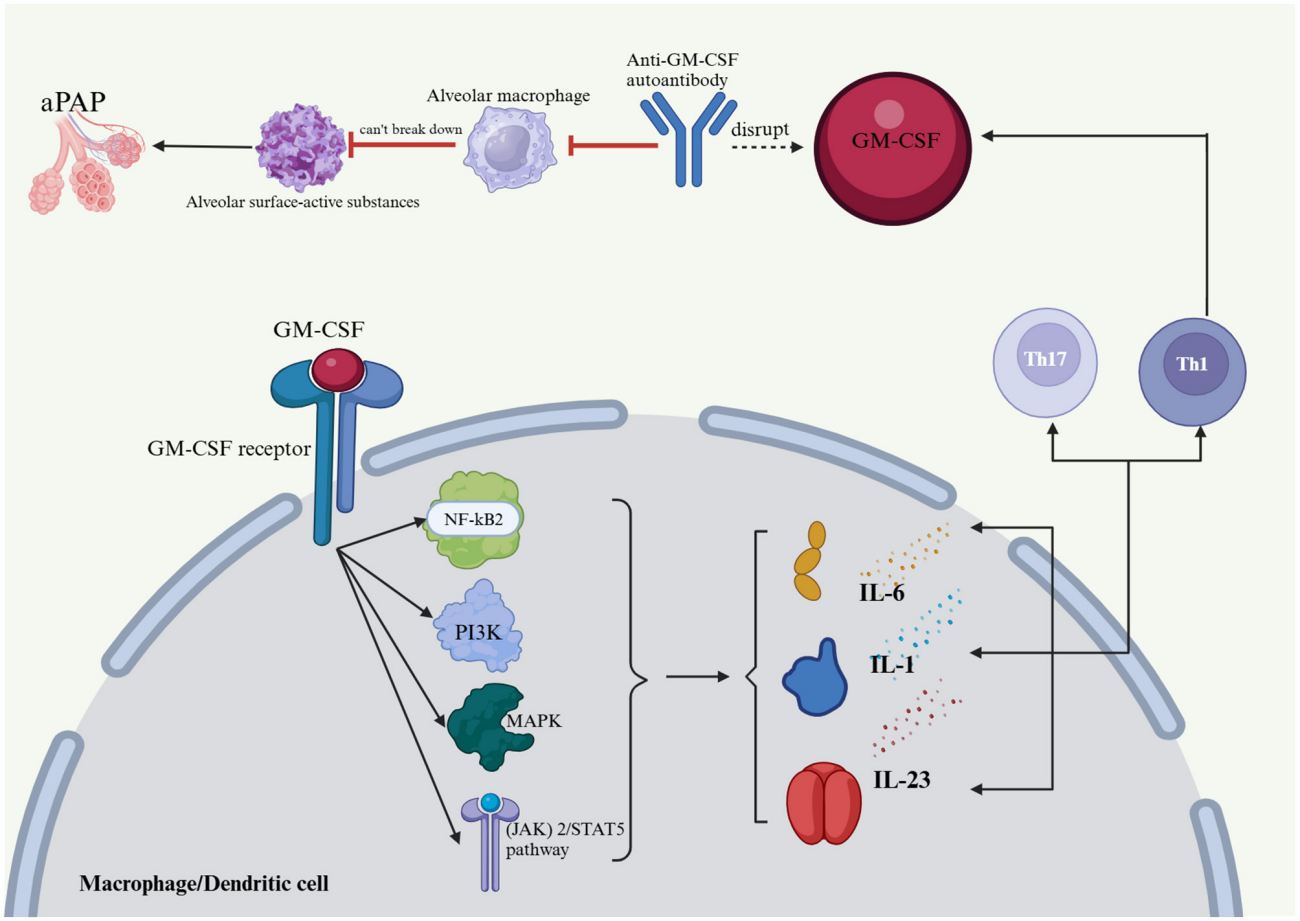
Effects of GM-CSF and anti-GM-CSF autoantibodies on the body and PAP formation. Anti-GM-CSF autoantibodies negatively affect alveolar macrophages, which in turn affects their ability to catabolize alveolar surface-active substances and protect the host from infectious diseases, which in turn leads to an accumulation of alveolar surface-active substances and PAP. The Janus kinase (JAK)2/STAT5, Ras-Raf-neutral gene-activated protein kinase (MAPK), nuclear factor (NF)-kB, and phosphatidylinositol 3-kinase (PI3K)-Akt pathways are implicated in GM-CSF receptor activation, and together, they regulate the immune effects of GM-CSF. GM-CSF activates macrophages and dendritic cells, which produce IL-23, IL-1, and IL-6 that drive Th17 and Th1 cell differentiation, thus creating a positive feedback loop in addition to the expression of GM-CSF by Th1 cells. PAP, pulmonary alveolar proteinosis; GM-CSF, granulocyte-macrophage colony-stimulating factor; IL-1, interleukin-1; IL-6, interleukin-6; IL-23, interleukin-23; Th1, T helper 1; Th17, T helper 17; (JAK) 2/STAT5 pathway, Janus kinase 2/signal transducer and activator of transcription 5; MAPK, mitogen-activated protein kinase; PI3K, phosphatidylinositol-3-kinase; NF-kB2, nuclear factor kappa-B.

GM-CSF plays a key role in immunomodulation (Figure 1), impacting bone marrow cell survival, proliferation, differentiation, and function. In inflammatory and autoimmune diseases, GM-CSF promotes bone marrow cell survival and proliferation, leading to increased monocyte, neutrophil, and macrophage numbers at sites of inflammation (22–24). GM-CSF is critical for dendritic cell survival, generation, and differentiation. It specifically promotes the differentiation and activation of mouse CD8+ splenic dendritic cells and human plasmacytoid dendritic cells, acting as a key CD8+ T-cell-derived “licensing factor” for mouse dendritic cell function (25–27). GM-CSF-activated macrophages and dendritic cells produce IL-23, IL-1, and IL-6, promoting Th17 and Th1 differentiation in a positive feedback loop. Th1 cells also express GM-CSF, amplifying the inflammatory response (28). GM-CSF-treated monocytes and macrophages typically display M1 polarization, which is linked to host defense and inflammation (29–31). GM-CSF receptor activation initiates several signaling pathways, including the JAK2/STAT5, Ras-Raf-MAPK, NF-κB, and PI3K-Akt pathways, which collectively mediate the immune effects of GM-CSF. Although GM-CSF is primarily proinflammatory, some evidence suggests that it may promote tolerogenic dendritic cell production, potentially mitigating the severity of autoimmune disease. Notably, GM-CSF enhances apoptotic cell uptake by macrophages through the EGF-like molecule lactadherin (32–34). Anti-GM-CSF autoantibodies impair alveolar macrophage function, hindering their ability to degrade surfactant and defend against infections. This can lead to surfactant buildup, progressive respiratory failure, and increased risk of infection, a condition known as PAP (1). The presence of anti-GM-CSF autoantibodies impairs the ability of GM-CSF to regulate *Mycobacterium tuberculosis*, increasing the susceptibility of macrophages to *M. tuberculosis* infection (35–37). Previous studies have established a close association between the production of anti-GM-CSF autoantibodies and genetic and hereditary factors (Figure 4) (38, 39).

There is currently no consensus on the optimal dosage, duration, or method of treatment for aPAP, largely due to the absence of standardized protocols and large-scale controlled trials. Future research should prioritize extensive, well-controlled studies to establish evidence-based treatment guidelines, including effective regimens, doses, durations, and delivery methods. Exploring personalized approaches could further improve therapeutic outcomes and management strategies.

Limitations of this study include reliance on data from public databases and websites in two main ways: this prevented the study from performing individualized matched analyses of patients receiving nebulized inhalation vs. subcutaneous GM-CSF. This methodological constraint may have introduced confounding variable bias, such as insufficient correction for baseline disease severity, comorbidities, and treatment cycle heterogeneity. Second, it also resulted in this study not further differentiating between sargramostim (yeast-derived glycosylated protein) and molgramostim (*E. coli*-derived non-glycosylated protein), which should have been further investigated to refine the study in terms of adverse effects, etc., even though their potency was almost identical. Additionally, our cohort consisted primarily of Asian individuals. Nonetheless, this study represents the largest retrospective analysis comparing the effects of GM-CSF inhalation and subcutaneous injection on aPAP, and we have made efforts to identify an appropriate dosage.

## Conclusion

Nebulized inhalation demonstrates greater efficacy and safety than does subcutaneous injection for aPAP treatment. The evidence suggests that the optimal dosing for nebulized inhalation peaks at 300–400 μg per day, whereas a treatment duration exceeding 24 weeks appears to yield the best results within this group. This approach not only improves therapeutic outcomes but also minimizes potential side effects associated with subcutaneous administration, making nebulized inhalation a compelling option for patient management in aPAP.
