# Corrigendum: Exogenous GM-CSF therapy for autoimmune pulmonary alveolar proteinosis: a systematic literature review

**DOI:** 10.3389/fmed.2025.1632193

**Published:** 2025-06-03

**Authors:** Wushu Chen, Xin Feng, Lun-kai Yao, Xingpei Li, Zhen-ming Yang, Xiu-yu Qin, Yu Li, Ye Qiu

**Affiliations:** ^1^Gastroenterology and Respiratory Internal Medicine Department, Guangxi Medical University Cancer Hospital, Nanning, Guangxi, China; ^2^State Key Laboratory of Respiratory Disease, National Clinical Research Center for Respiratory Disease, Guangzhou Institute of Respiratory Health, The First Affiliated Hospital of Guangzhou, The First Affiliated Hospital of Guangzhou Medical University, Guangzhou, China; ^3^Medical Oncology of Respiratory Medicine, Guangxi Medical University Cancer Hospital, Nanning, Guangxi, China; ^4^Department of Respiratory and Critical Medicine, Yiyang Central Hospital, Yiyang, Hunan, China

**Keywords:** GM-CSF, aPAP, inhalation, subcutaneous injection, treatment dosage and duration

In the published article, the order of affiliations 1 and 2 was incorrect, “Gastroenterology and Respiratory Internal Medicine Department, Guangxi Medical University Cancer Hospital, Nanning, Guangxi, China” should be affiliation 1 and “State Key Laboratory of Respiratory Disease, National Clinical Research Center for Respiratory Disease, Guangzhou Institute of Respiratory Health, The First Affiliated Hospital of Guangzhou, The First Affiliated Hospital of Guangzhou Medical University, Guangzhou, China” should appear as affiliation 2.

In the published article, there was an error regarding the affiliation for Wushu Chen. As well as having affiliation 1, they should also have affiliation 2.

In the published article, the order of the corresponding authors in the correspondence section has been updated to:

Ye Qiu, yeqiu2013graduated@163.com

Yu Li, ly.20052008@163.com

The authors apologize for this error and state that this does not change the scientific conclusions of the article in any way. The original article has been updated.

