# Supplementary material for: Exogenous GM-CSF therapy for autoimmune pulmonary alveolar proteinosis: a systematic literature review
Source: Front Med (Lausanne). 2025 May 8;12:1552566. doi: 10.3389/fmed.2025.1552566 (PMC12094945; doi:10.3389/fmed.2025.1552566)
Supplement: Supplementary file 1 [file Table_1.DOCX]

Contents

[Methods 1](#_Toc361371571)

[**1.1 Data Processing Considerations 1**](#_Toc488240081)

[Results 1](#_Toc316026180)

[**2.1 Supplement Material Table 1 pre- and post-treatment baseline values across the different groups 1**](#_Toc724948229)

[**2.2 Supplement Material Table 2 GM-CSF treatment cycle statistics 3**](#_Toc1530155372)

[**2.3 Supplement Material Table 3 GM-CSF treatment cycle duration statistics 4**](#_Toc1204664379)

[**2.4 Supplement Material Table 4 GM-CSF treatment cycle duration efficacy statistics 4**](#_Toc318393937)

[**2.5 Supplement Material Table 5 GM-CSF administration dose statistics 5**](#_Toc1865134482)

[**2.6 Supplement Material Table 6 GM-CSF administration dose duration statistics 6**](#_Toc496443715)

**Methods**

**1.1 Data Processing Considerations**

When calculating whether the difference between the indicators before and after the treatment is significant or not, because the number of data before and after the treatment is different, we matched the data and eliminated the outliers so that the number of values before and after the treatment is the same and then carried out a paired test to find the P value.

**Results**

**2.1 Supplement Material Table 1 Differences between** **pre- and posttreatment baseline values across the different groups**

| **Metrics** | **Subcutaneous injection group** | **Nebulized inhalation group** | **P value** |
| --- | --- | --- | --- |
| **PaO2 (mmHg)** | 18.63±9.16 | 11.78±5.27 | <0.001 |
| **PaCO2 (mmHg)*** | -17.59* | -7.50±2.55 | 0.667 |
| **SaO2** | 19.00±8.00% | 4.05* | 0.500 |
| **FVC baseline** | 9.57±6.46% | 5.01±6.93% | 0.067 |
| **FEV1*** | 21.33%* | 3.24±5.86% | 0.054 |
| **TLC*** | 17%* | 26.52±8.59% | 0.571 |
| **VC*** | 44%* | 4.59±1.98% | 0.015 |
| **DLCO** | 28.00±7.07% | 8.43±4.27% | <0.001 |
| **(A-a) O2 baseline(mmHg)** | -25.55±3.50 | -13.16±3.25 | <0.001 |

*For the labelled indicators, one of the sets of pre- and posttreatment data had only one case; thus, we averaged the other set of data and compared it to the former.

**2.2 Supplement Material Table 2 GM-CSF treatment cycle duration statistics**

| **GM-CSF Treatment Cycle Duration** | **Effective** | **Ineffective** | **P value** |
| --- | --- | --- | --- |
| **Subcutaneous injection** | 22 | 22 | 0.586 |
| **<=12 w** | 5 | 2 |  |
| **12 w~24 w** | 5 | 7 |  |
| **>24 w** | 12 | 13 |  |
| **Nebulized inhalation** | 54 | 35 | 0.031 |
| **<=12 w** | 9 | 6 |  |
| **12 w~24 w** | 30 | 27 |  |
| **->24w** | 22 | 3 |  |

**2.3 Supplement Material Table 3 GM-CSF Treatment Cycle Duration Efficacy Statistics**

| **GM-CSF Treatment Cycle Duration** | **Subcutaneous injection（%）** | **Nebulized inhalation（%）** | **P value** |
| --- | --- | --- | --- |
| **<=12 w** | 71.43 | 60 | 0.102 |
| **12 w~24 w** | 41.67 | 52.63 | 0.119 |
| **>24 w** | 48 | 88 | <0.001 |

**2.4 Supplement Material Table** **4 GM-CSF administration dose duration statistics**

| **GM-CSF administration dose** | **Effective** | **Ineffective** | **P value** |
| --- | --- | --- | --- |
| **Subcutaneous injection** | | | |
| **<=400 mg/day** | 13 | 11 | 0.795 |
| **>400 mg/day** | 23 | 17 |  |
| **<=300 μg/day** | 12 | 10 | 0.842 |
| **>300 μg/day** | 24 | 18 |  |
| **Nebulized inhalation** | | | |
| **<=400 mg/day** | 146 | 47 | 0.036 |
| **>400 mg/day** | 14 | 11 |  |
| **<=300 μg/day** | 144 | 47 | 0.076 |
| **>300 μg/day** | 16 | 11 |  |
